# Seroprevalence of Infection-Induced SARS-CoV-2 Antibodies — United States, September 2021–February 2022

**DOI:** 10.15585/mmwr.mm7117e3

**Published:** 2022-04-29

**Authors:** Kristie E.N. Clarke, Jefferson M. Jones, Yangyang Deng, Elise Nycz, Adam Lee, Ronaldo Iachan, Adi V. Gundlapalli, Aron J. Hall, Adam MacNeil

**Affiliations:** ^1^CDC COVID-19 Emergency Response Team; ^2^ICF International, Rockville, Maryland.

In December 2021, the B.1.1.529 (Omicron) variant of SARS-CoV-2, the virus that causes COVID-19, became predominant in the United States. Subsequently, national COVID-19 case rates peaked at their highest recorded levels.* Traditional methods of disease surveillance do not capture all COVID-19 cases because some are asymptomatic, not diagnosed, or not reported; therefore, the proportion of the population with SARS-CoV-2 antibodies (i.e., seroprevalence) can improve understanding of population-level incidence of COVID-19. This report uses data from CDC’s national commercial laboratory seroprevalence study and the 2018 American Community Survey to examine U.S. trends in infection-induced SARS-CoV-2 seroprevalence during September 2021–February 2022, by age group.

The national commercial laboratory seroprevalence study is a repeated, cross-sectional, national survey that estimates the proportion of the population in 50 U.S. states, the District of Columbia, and Puerto Rico that has infection-induced antibodies to SARS-CoV-2.^†^ Sera are tested for anti-nucleocapsid (anti-N) antibodies, which are produced in response to infection but are not produced in response to COVID-19 vaccines currently authorized for emergency use or approved by the Food and Drug Administration in the United States.^§^

During September 2021–February 2022, a convenience sample of blood specimens submitted for clinical testing was analyzed every 4 weeks for anti-N antibodies; in February 2022, the sampling period was <2 weeks in 18 of the 52 jurisdictions, and specimens were unavailable from two jurisdictions. Specimens for which SARS-CoV-2 antibody testing was ordered by the clinician were excluded to reduce selection bias. During September 2021–January 2022, the median sample size per 4-week period was 73,869 (range = 64,969–81,468); the sample size for February 2022 was 45,810. Seroprevalence estimates were assessed by 4-week periods overall and by age group (0–11, 12–17, 18–49, 50–64, and ≥65 years). To produce estimates, investigators weighted jurisdiction-level results to population using raking across age, sex, and metropolitan status dimensions from 2018 American Community Survey data^¶^ (*1*). CIs were calculated using bootstrap resampling (*2*); statistical differences were assessed by nonoverlapping CIs. All specimens were tested by the Roche Elecsys Anti-SARS-CoV-2 pan-immunoglobulin immunoassay.** All statistical analyses were conducted using R statistical software (version 4.0.3; The R Foundation). This activity was reviewed by CDC, approved by respective institutional review boards, and conducted consistent with applicable federal law and CDC policy.^††^

During September–December 2021, overall seroprevalence increased by 0.9–1.9 percentage points per 4-week period. During December 2021–February 2022, overall U.S. seroprevalence increased from 33.5% (95% CI = 33.1–34.0) to 57.7% (95% CI = 57.1–58.3). Over the same period, seroprevalence increased from 44.2% (95% CI = 42.8–45.8) to 75.2% (95% CI = 73.6–76.8) among children aged 0–11 years and from 45.6% (95% CI = 44.4–46.9) to 74.2% (95% CI = 72.8–75.5) among persons aged 12–17 years (Figure). Seroprevalence increased from 36.5% (95% CI = 35.7–37.4) to 63.7% (95% CI = 62.5–64.8) among adults aged 18–49 years, 28.8% (95% CI = 27.9–29.8) to 49.8% (95% CI = 48.5–51.3) among those aged 50–64 years, and from 19.1% (95% CI = 18.4–19.8) to 33.2% (95% CI = 32.2–34.3) among those aged ≥65 years.

**FIGURE:**
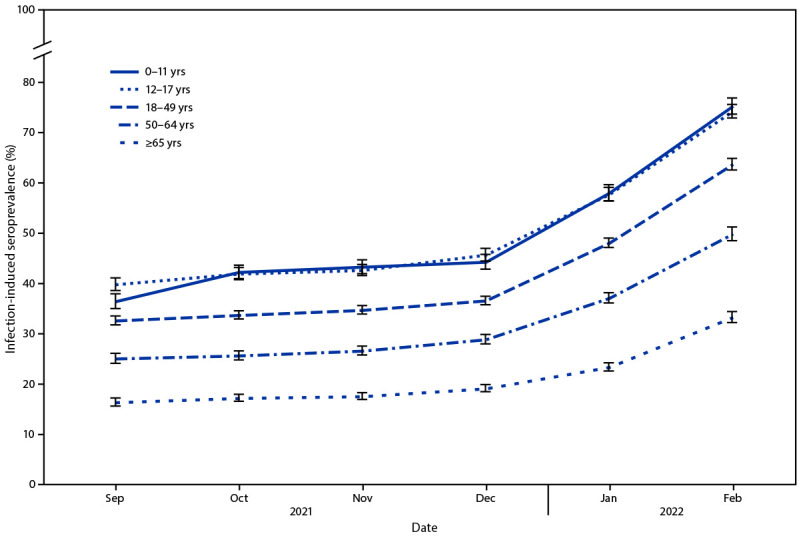
Seroprevalence of infection-induced SARS-CoV-2 antibodies,* by age group — United States, September 2021–February 2022 * Error bars represent 95% CIs at each time point.

The findings in this report are subject to at least four limitations. First, convenience sampling might limit generalizability. Second, lack of race and ethnicity data precluded weighting for these variables. Third, all samples were obtained for clinical testing and might overrepresent persons with greater health care access or who more frequently seek care. Finally, these findings might underestimate the cumulative number of SARS-CoV-2 infections because infections after vaccination might result in lower anti-N titers,^§§^^,^^¶¶^ and anti-N seroprevalence cannot account for reinfections.

As of February 2022, approximately 75% of children and adolescents had serologic evidence of previous infection with SARS-CoV-2, with approximately one third becoming newly seropositive since December 2021. The greatest increases in seroprevalence during September 2021–February 2022, occurred in the age groups with the lowest vaccination coverage; the proportion of the U.S. population fully vaccinated by April 2022 increased with age (5–11, 28%; 12–17, 59%; 18–49, 69%; 50–64, 80%; and ≥65 years, 90%).*** Lower seroprevalence among adults aged ≥65 years, who are at greater risk for severe illness from COVID-19, might also be related to the increased use of additional precautions with increasing age (*3*).

These findings illustrate a high infection rate for the Omicron variant, especially among children. Seropositivity for anti-N antibodies should not be interpreted as protection from future infection. Vaccination remains the safest strategy for preventing complications from SARS-CoV-2 infection, including hospitalization among children and adults (*4**,**5*). COVID-19 vaccination following infection provides additional protection against severe disease and hospitalization (*6*). Staying up to date^†††^ with vaccination is recommended for all eligible persons, including those with previous SARS-CoV-2 infection.

## Footnotes

*
https://covid.cdc.gov/covid-data-tracker/#trends_dailycases

†
https://www.cdc.gov/coronavirus/2019-ncov/cases-updates/commercial-lab-surveys.html

§
https://www.fda.gov/emergency-preparedness-and-response/coronavirus-disease-2019-covid-19/covid-19-vaccines

¶
https://www.census.gov/programs-surveys/acs/data.html

**
https://www.fda.gov/media/137605/download

†† 45 C.F.R. part 46, 21 C.F.R. part 56; 42 U.S.C. Sect. 241(d); 5 U.S.C. Sect. 552a; 44 U.S.C. Sect. 3501 et seq.

§§
https://www.medrxiv.org/content/10.1101/2022.04.18.22271936v1?rss

¶¶
https://www.medrxiv.org/content/10.1101/2021.10.25.21264964v1

*** https://covid.cdc.gov/covid-data-tracker/#vaccination-demographics-trends (accessed April 17, 2022).

†††
https://www.cdc.gov/coronavirus/2019-ncov/vaccines/stay-up-to-date.html
